# Psychometric Properties of Chosen Scales Evaluating Disability in Low Back Pain—Narrative Review

**DOI:** 10.3390/healthcare12111139

**Published:** 2024-06-03

**Authors:** Bartosz Chmielewski, Maciej Wilski

**Affiliations:** 1 Department of Health Sciences, State University of Applied Sciences in Konin, 62-510 Konin, Poland; bartchmiel@wp.pl; 2 Department of Adapted Physical Activity, Poznan University of Physical Education, ul. Królowej Jadwigi 27/39, 61-871 Poznan, Poland

**Keywords:** disability, low back pain, scales, questionnaires

## Abstract

Low back pain (LBP) is one of the most common disabling conditions. This disability significantly reduces the quality of life of LBP patients. This article reviews the most common and well-known measures currently used to assess disability in LBP, such as the Oswestry Disability Index (ODI), the Roland–Morris Disability Questionnaire (RMDQ), the Quebec Back Pain Disability Scale (QBPDS), the Low Back Outcome Score (LBOS), and the Low Back Pain Rating Scale (LBPRS). To reliably evaluate questionnaires and other measurement methods, there are parameters known as psychometric properties, which consist primarily of the validity, reliability and sensitivity. These methods are based on a multi-item questionnaire assessing physical functioning that is completed independently by the patient. They can be used to assess the disability associated with many conditions. All are specific to LBP, and their psychometric properties have been tested on a relevant population of patients with the condition and published in peer-reviewed publications.

## 1. Introduction

Low back pain (LBP) crosses cultural boundaries and is a common and debilitating condition worldwide [1]. It is estimated that approximately half of all adults will experience spinal pain at some point in their lives, making it one of the most common conditions [2]. In addition, a staggering 80% of adults will experience at least one pain-related event in their lifetime [2]. Within the spectrum of spinal disorders, LBP is polyetiological, suggesting multiple causative factors. While some cases may be transient, a significant proportion tend to recur and become chronic [2]. The impact goes beyond physical discomfort, with financial burdens resulting from medical expenses, absenteeism from work, and possible early retirement. In addition, the psychological toll of spinal pain cannot be underestimated [2,3]. As a result, people living with spinal pain experience a gradual decline in both their physical fitness and quality of life. The recurrent nature of this pain undermines daily functioning, leading to limitations in social engagement, work productivity, and recreational pursuits.

In addressing the decreasing functionality of individuals with LBP [4], there is significant interest in not only quantifying pain levels or measuring patients’ quality of life [5], but also in assessing the degree of disability. These measures serve purposes beyond mere epidemiologic documentation, extending into the realm of treatment evaluation and comparison, whether in clinical practice or research settings [6]. Critically, disability assessment scales for LBP must meet the standards of evidence-based medicine. To meet these criteria, large-scale multicenter clinical trials with diverse patient cohorts are essential. Methodological rigor in group selection, including precise inclusion and exclusion criteria, as well as randomized allocation to treatment and control arms, is paramount. In addition, ensuring double blinding, in which both the patient and the investigator are unaware of the treatment allocation, coupled with extended follow-up periods, enhances the validity of study results [5]. Adherence to these rigorous standards facilitates the identification of causal relationships between treatment modalities and their efficacy, allowing for more informed decisions regarding treatment parameters [7]. Thus, the robust methodologies underpinning disability assessment tools contribute significantly to the advancement of therapeutic approaches for LBP, benefiting patients and practitioners alike.

In the evaluation of questionnaires and measures, attention is focused on psychometric properties, which include relevance, reliability, and sensitivity. Relevance examines whether a test effectively measures what it purports to measure and ensures its applicability in specific clinical contexts, such as assessing disability in sacral pain. Reliability refers to the consistency, reproducibility, and freedom from measurement error of the results obtained from a questionnaire, assuming constancy in the assessment of disability. Sensitivity, on the other hand, describes the ability of a test to detect differences in the disability levels between patients or groups. An ideal test should detect differences between groups with a reasonably moderate sample size, making it more sensitive and requiring smaller cohorts for statistically valid assessments. Sensitivity to change, also known as responsiveness, measures a test’s ability to detect clinically significant changes in disability levels within the same patient and is thus distinct from conventional sensitivity [8].

This article reviews the common and well-established measures used to assess disability in LBP. Notable methods include the Oswestry Disability Index (ODI), Roland–Morris Disability Questionnaire (RMDQ), Quebec Back Pain Disability Scale (QBPDS), Low Back Outcome Score (LBOS), and Low Back Pain Rating Scale (LBPRS). Each of these tools provides a unique insight into the assessment of disability associated with LBP and contributes to the comprehensive understanding and management of this prevalent condition.

## 2. Methods Review

### 2.1. The Oswestry Disability Index

The Oswestry Disability Index (ODI) is one of the most prominent measures used to assess the level of disability in individuals with sacral pain [9]. The questionnaire, which originated in 1976, was presented to the medical community at the International Society for the Study of the Lumbar Spine conference in Paris shortly after its inception. Over the years, the ODI has undergone numerous revisions and translations into various languages to improve its utility and applicability to diverse populations. The design of the ODI reflects a deliberate emphasis on assessing physical activity rather than addressing the psychological impact of acute or chronic pain [10]. Recognizing the complexities inherent in defining and measuring disability (which encompasses impairment, disability, and handicap), the ODI’s focus on physical functionality serves to provide a comprehensive assessment framework for individuals coping with sacral pain.

The ODI questionnaire consists of 10 sections, 8 of which focus on activities of daily living (ADL) and 2 of which focus on pain assessment [11,12,13]. The ADL sections include tasks such as personal care (washing, dressing), lifting heavy objects, walking, sitting, standing, sleeping, traveling, and social activities. In contrast, the pain-related sections assess the intensity and changes in pain levels over time. Each question within the ODI has six possible answers, which are scored from 0 to 5. Thus, the highest possible score is 50, while the lowest score is 0. These individual scores are then aggregated to produce a total score. The resulting score can be expressed either on a point scale from 0 to 50 or on a percentage scale from 0% to 100%. Interpretation of the ODI score provides a nuanced understanding of the patient’s functional status. A score of 0 to 4 indicates no disability, while 5 to 14 indicates mild disability. Moderate disability falls within the range of 15 to 24 points, while severe disability includes scores of 25 to 34 points. A score of 35 points or more indicates extreme suffering and disability [10,11,12,13].

A validation analysis of the ODI has been conducted, including translations and comparisons with pain-related questionnaires such as the Visual Analog Scale (VAS), the McGill Pain Questionnaire, and the Pain Disability Index [9,10]. According to reports and psychometric evaluations, the ODI has good construct validity and acceptable internal consistency. It typically takes less than 5 min to complete and less than 1 min to obtain results. Originally used primarily in populations with chronic and severe disabilities, the ODI has proven to be a valuable tool for assessing milder impairments [14]. Nevertheless, there are instances where the ODI shows limited effectiveness in measuring patient satisfaction after discectomy. The authors point out additional factors that may influence the results of the measurement, such as the severity of the hernia, the surgical approach, or the preoperative health status [15].

### 2.2. Roland–Morris Disability Questionnaire

Another equally notable and widely used method for assessing disability in LBP is the Roland–Morris Disability Questionnaire (RMDQ) [16]. This questionnaire has undergone several iterations, resulting in different versions such as RM-12, RM-16, RM-18, RM-23, SIP-RM, MRMQ (a modified version that assesses functional limitations over the past four weeks), and RMDQ.att (adapted to measure attitudes toward back pain and disability—a spouse-completed version). The RMDQ serves as a scale for quantifying disability, with higher scores on a 24-point scale corresponding to greater levels of disability. The questionnaire includes a number of questions, including those related to basic household activities such as bending, kneeling, standing, dressing, housework, and climbing stairs. It also includes questions about pain-related behaviors, such as avoiding leaving the house, limiting daily activities, sleep disturbances, and irritability [17].

In administering the RMDQ, the patient is instructed to mark each relevant statement with which he or she agrees, and the total score is derived by summing the marked statements. Clinical progress over time is assessed by comparing the results of serial questionnaires. For example, if a patient’s initial score was 12 at the start of treatment and was reduced to 2 at the end of treatment, indicating a 10-point improvement, the percentage of improvement would be calculated as 83% (10/12 × 100). However, concerns have been raised about the sensitivity and susceptibility of the 24-point scale to measurement error [16]. Studies have suggested that an improvement in patients with baseline scores below 4 RMDQ points and worsening in patients with baseline scores above 20 RMDQ points may not be reliable indicators of true clinical change. These findings caution against over-reliance on the 24-point scale and emphasize the need for the careful interpretation of results, taking into account individual patient characteristics and potential measurement limitations.

The RMDQ is proving to be an invaluable, adaptable and simple tool for assessing the disability associated with LBP. With an average completion time of approximately five minutes, it offers efficiency in the clinical setting. The RMDQ has been translated into several languages [18], with the Polish version being a notable example. This translation exemplifies the successful adaptation of the questionnaire to different linguistic contexts while maintaining its psychometric properties [17].

In the case of the Polish version, a thorough analysis confirms its reliability and validity, mirroring the psychometric properties of the original English version. Furthermore, the strong correlations observed between the Polish RMDQ and scales such as the SF-36 Physical Component Summary (PCS) and pain scales underscore its utility in assessing the functional status of Polish-speaking individuals with sacral pain. This successful translation of the RMDQ into Polish highlights its versatility and applicability across different cultural and linguistic settings. It not only facilitates cross-cultural research, but also increases the accessibility of this valuable tool to clinicians and researchers worldwide [17].

### 2.3. Quebec Back Pain Disability Scale

Another widely used measure of the disability associated with back pain is the Quebec Back Pain Disability Scale (QBPDS). Originally described by Kopeć et al. in 1995 [19] the final version of the QBPDS items was selected through a rigorous test–retest reliability study [20]. The scale consists of 20 daily activities, each of which is rated by the patient for its difficulty on a scale from 0 (“not at all difficult”) to 5 (“impossible to do”). The cumulative scores for all items yield a total score ranging from 0 to 100, with higher scores indicating greater levels of disability.

These activities are grouped into six categories corresponding to the different areas potentially affected by back pain: bed and rest (questions 1–3), sitting and standing (questions 4–6), walking (questions 7–9), moving (questions 10–12), bending and stooping (questions 13–16), and handling heavy objects (questions 17–20). Patients are instructed to rate their ability to perform each activity on the day they complete the questionnaire. The QBPDS questionnaire typically takes up to 5 min to complete [20,21,22].

Kopeć et al. conducted a comprehensive assessment of the reliability and validity of the QBPDS and compared it with other disability scales [19]. Their study included calculating the test–retest and internal consistency coefficients, assessing the construct validity, and evaluating the responsiveness to change, as indicated by a global index. In addition, direct comparisons were made with well-known scales such as the RMDQ, ODI and SF-36. The test–retest reliability of the QBPDS scale was found to be 0.92, indicating strong consistency over time. In addition, the Cronbach’s alpha coefficient, a measure of internal consistency, was calculated to be 0.96, indicating high reliability. As expected, the QBPDS scale showed correlations with other measures of disability, pain, and sociodemographic characteristics. Based on their findings, Kopeć et al. recommended the QBPDS scale as a valuable outcome measure for clinical trials and for monitoring the progress of individual patients undergoing treatment or rehabilitation programs [19].

Other studies and critical analyses examining reports using the QBPDS have consistently found strong correlations with other established measures such as the RMDQ and the ODI. In particular, the QBPDS scale has demonstrated remarkable sensitivity, with Receiver Operating Characteristic (ROC) values consistently above 0.74 and often above 0.80. In addition, studies have reported no floor or ceiling effects, indicating the ability of the scale to capture a wide range of disability levels [23].

However, caution should be exercised when using the QBPDS to assess disability in patients with nonspecific LBP. Despite its widespread use, there remains a lack of robust evidence supporting all measurement properties for any version of the QBPDS scale. Thus, while the QBPDS scale may provide valuable insight into disability assessment in certain contexts, its use in patients with nonspecific LBP should be approached cautiously, taking into account potential limitations and the need for further validation studies [22].

### 2.4. The Low-Back Outcome Scale

The Low Back Outcome Scale (LBOS), developed by Greenough and Fraser, is a less well-known questionnaire designed to assess the level of disability associated with LBP [24,25]. This scale consists of 13 factors that collectively capture different aspects of disability perception. The first factor relates to the current level of pain, which is assessed using the Visual Analog Scale (VAS) and categorized as follows: 7–10 (0 points), 5–6 (3 points), 3–4 (6 points), and 0–2 (9 points). Employment status is another factor, with scoring based on patients’ ability to work: no work due to pain (0 points), part-time work (3 points), full-time work with restrictions (6 points), and full-time work without restrictions (9 points). Similarly, the ability to perform household chores or odd jobs is assessed, ranging from unable to perform (0 points) to no difficulty (9 points).

The questionnaire also includes assessments of involvement in sports and social activities, ranging from none (0 points) to full involvement (9 points), and need for rest, ranging from more than half a day (0 points) to no need for rest (6 points). Medical visits, pain medication use, and sexual activity are rated on a scale of 0–2–4–6, while quality of sleep, walking, sitting, traveling, and dressing are rated on a scale of 0–1–2–3 [26]. The LBOS scale ranges from 0 to 75, with higher scores indicating better performance. It classifies patients according to a 4-point grading scheme: excellent ≥65; good 50–64; fair 30–49; and poor 0–29 [25].

The LBOS has demonstrated reliability and good internal consistency. The minimum clinically important difference (MCID) is reported to be 7.5 points, and it correlates well with the ODI (r = 0.87). While the LBOS is straightforward to administer, any weighting should be approached with caution. The LBOS encompasses all aspects of the International Classification of Functioning, Disability, and Health (ICF), although slight extensions of the questionnaire have been noted. However, a ceiling effect has been reported in populations with uncompensated LBP. Given the comprehensive nature of the ICF dimensions, the summation of all items to derive a total score may lead to item masking. Nevertheless, this multidimensional approach provides a holistic assessment of the patient and is a strength of the LBOS. There is potential for item weighting bias and cross-contamination bias. Therefore, it is recommended that subscores are used for each dimension (pain, functional, and ability items) rather than relying solely on the total score [27].

### 2.5. The Low Back Pain Rating Scale

The Low Back Pain Rating Scale (LBPRS) is another notable tool for assessing LBP [28]. It consists of three distinct components: pain, disability, and physical impairment. The pain component consists of six questions divided into two groups: three questions focusing on back pain and three questions focusing on lower extremity pain. Each question is scored on a Visual Analog Scale (VAS) ranging from 0 to 10 points. Measurements include the current LBP/lower extremity pain intensity, the worst LBP/lower extremity pain experienced in the past two weeks, and the average level of back pain/lower extremity pain over the same period. The final score for the pain component ranges from 0 to 30 for both back and lower extremity pain, with a total possible score of 60.

The disability component consists of 15 questions that assess the patient’s ability to perform various daily activities such as sleeping, household chores, walking, sitting, lifting objects, working, dressing, driving, walking, getting out of a chair, climbing stairs and social interactions, and the patient’s expectations about future pain. Each question is scored on a three-point Likert scale and can be answered “yes” (0 points), “may be a problem” (1 point), and “no” (2 points). The disability component results in a total score of 0–30 [28].

The physical impairment component is assessed by measuring patients’ back muscle strength, spinal mobility, mobility, and pain medication use. Muscle strength and back/patient mobility are assessed with a specific physical examination and each is scored on a scale of 0–10. Pain medication use is assessed using the following scale: “used no medication during the week” (0 points); “used NSAIDs/non-narcotic analgesics up to 4 times per week” (2 points); “used NSAIDs/non-narcotic analgesics more than 4 times per week” (4 points); “used morphine up to 4 times per week” (8 points); and “used morphine more than 4 times per week” (10 points). The physical impairment component ranged from 0 to 40 points. Thus, the listed components include the following: 60 points for pain, 30 points for disability, and 40 points for physical impairment. The final LBPRS score ranges from 0 (in patients without back problems) to 130 (in a disabled patient) [13,21,28].

The LBPRS questionnaire can be completed in approximately 15 min. The results are typically calculated in 3–5 min. In addition, the rating scale can be adapted for in-office, telephone, and mailed administration with minor modifications that have minimal impact on the information collected. Despite its limitations, such as the small number of patients recruited for validation, the study supports the use of the LBPRS scale for the functional assessment of patients with LBP. Its versatility and ease of administration make it a practical tool for both clinical practice and research, helping to assess and monitor the functional status of patients with LBP [13,21,28].

## 3. Discussion

Although there are many questionnaires and scales for assessing LBP, some of the most commonly used in clinical and research settings include the ODI, RMDQ, QBPDS, LBOS, and LBPRS [13]. A comparison of the basic characteristics of the selected scales is given in Table 1.

At the outset, it should be emphasized that research clearly indicates the importance of differentiating patients based on the causes of low back pain (LBP), as well as the nature of their work and psychosocial functioning [41]. Low back pain is a complex and multifactorial condition, often influenced by various physical, biomechanical, psychosocial, and occupational factors. Therefore, understanding the specific factors contributing to an individual’s LBP is crucial for effective assessment and management.

Taking into account the validation analysis of the aforementioned questionnaires, the ODI demonstrates adequate content validity by addressing the daily activities typically encountered by people with LBP. However, it does not include activities related to sport, leisure or work. The ODI is easy to administer and calculate. It has a strong correlation with other scales such as the McGill Pain Questionnaire (MPQ), the Visual Analog Scale (VAS), and the SF-36. Although a floor effect has been observed, there is no evidence of a ceiling effect [21,23].

The RMDQ is also easy to administer and provides a quick assessment. The RMDQ assesses only a limited range of pain-related problems during the performance of daily activities. It includes a small number of psychosocial items that may not be directly related to pain-related functional limitations (such as appetite or irritability). The RMDQ has a strong correlation with other instruments such as the ODI, SF-36, and QBPDS regarding its physical activity assessment. The responsiveness depends on the level of disability [21,23].

The RMDQ has been shown to provide reliable measures that are useful in inferring levels of disability and to be sensitive to changes over time in groups of patients with LBP. However, there are reports suggesting that the 24-point scale may be too sensitive and prone to measurement error [16]. According to Yao and colleagues [30], the ODI is superior in its validity and reliability for cross-sectional surveys, making it particularly applicable. Conversely, in the context of intervention studies, the RMDQ comes to the fore due to its superior responsiveness, which is particularly evident in patients with lumbar disc herniation.

The QBPDS questionnaire is also characterized by its ease of administration and scoring. The validity and content of the QBPDS questionnaire are well regarded because it includes different types of activities selected by patients and health professionals, and has good measurement properties. However, it lacks a question on sexual activity, which may be important to some researchers. The questionnaire also provides the opportunity to differentiate patient groups based on their level of disability and self-reported health status [21,23].

The LBOS questionnaire incorporates several dimensions of the ICF, so summing to obtain a total score may lead to item masking. Nevertheless, the inclusion of different dimensions of the ICF provides a multidimensional assessment of the patient and is a strength of the LBOS. According to Smeets et al. [21], construct validity testing confirmed the conditional independence of the LBPRS from physician assessment, controlling for patient assessment (*p* 0.00005), and the conditional independence of the LBPRS and patient assessment, controlling for physician assessment (*p* 0.00005); this suggests that the scale correlates with both the physician global assessment and patient global assessment.

The analysis of the psychometric properties of the scales presented inevitably involves a consideration of the measurement of their reliability. It has been shown that the test–retest reliability of the ODI is high. The values range from r = 0.73 to 0.99 and vary according to the time interval between measurements. The longer the measurement interval, the lower the score. The intraclass correlation coefficient ranges from 0.84 to 0.94 [14,23].

The RMDQ has been shown to have good internal consistency (Cronbach’s alpha ranging from 0.84 to 0.96). Its reliability is higher for short time intervals (1–14 days) than for longer time intervals (>6 weeks). The Pearson correlation coefficient for the test–retest reliability in patients with acute and subacute LBP is 0.91 for the same day, 0.88 for 1 week, and 0.83 for 3 weeks. For patients with chronic LBP, the coefficient was 0.72 (2–6 months) [21,23].

The test–retest reliability for the QBPDS was reported to be from 0.84 to 0.93 [21,35], while the Cronbach’s alpha coefficient was in general higher than 0.9 [19,21]. The scale correlated as expected with other measures of disability, pain, medical history, use-related variables, work-related variables, and sociodemographic characteristics. Significant changes in disability over time were observed, as well as differences in the change scores between patients, where differences in the direction of change would be expected [19].

The reliability of the LBOS was also analyzed. A Cronbach’s alpha coefficient of 0.77 and above was obtained from the internal consistency test performed on the various studies. The overall test–retest reliability agreement was 84%, with a reliability coefficient (K) ranging from 0.34 to 0.99 (*p* < 0.05), depending on the translation of the scale [36,37,38,39,40]. A Bland–Altman plot was calculated, showing that only 5% of the patients’ scores changed by more than 11.6 points on the scale between the test and the retest, which is not sufficient to change the outcome categories [29]. The reliability of the LBPRS scale is also highly rated (inter-regional reliability—97.7%) [28,29]. However, there is a lack of information on the minimum detectable change or the standard error of measurement (SEM) of the scale, and confidence intervals have not been provided [21].

Comparison with other disease-specific outcome measures such as the RMDQ and the QBPDS, as well as comparison with a general health questionnaire such as the Short Form-36 (SF-36), yields comparable results in terms of validity, reliability, and response time. The test–retest reliability is slightly higher for the ODI compared with the RMDQ and the QBPDS. The responsiveness is higher for the ODI than for the QBPDS. The ODI appears to have a slight advantage in assessing patients with chronic and more severe disability, and is more sensitive to improvement compared to patients with no change [14]. The comparative studies by Lauridsen and colleagues [28] clearly showed that all analyzed pain measures (ODI, RMDQ, QBPDS and LBPRS) had similar responsiveness, which in turn was comparable to the disability measures. However, the receiver operating characteristic (ROC) curve for the LBPRS—Pain domain consistently outperformed the ROC for LBPRS—Disability, which was considered inconclusive for responsiveness. In the case of the LBPRS, the disability domain shows a weaker response compared to the RMDQ and ODI. There is also a lack of information on important psychometric properties such as the minimal detectable change (MDC) and SEM [21].

## 4. Conclusions

A questionnaire used to assess disability should have adequate sensitivity and reliability. However, these are not sufficient conditions to determine its full validity. The validation of the test in the target population, in a specific clinical situation such as chronic LBP, allows its validity in this condition to be estimated and subsequent inferences about its utility in research or clinical practice to be made. The described tests are easy to administer and calculate.

The ODI is a simple and well-analyzed questionnaire that is widely used in comparative studies. However, it is not recommended for evaluating preventive interventions because of the floor effect. The RMDQ is a good tool for assessing LBP in conjunction with a general health assessment and can be used successfully for mild to moderate generalized LBP. The QBPDS is well targeted for disability assessment and allows for consistent responses, making it an excellent tool for disability assessment. When used in conjunction with an independent pain assessment tool, the QBPDS may be recommended for the assessment of LBP. The LBOS is useful because it is short, covers important treatment outcomes, and clearly distinguishes between pain and disability. Therefore, the LBOS may be suggested for studies where only a brief assessment is needed (e.g., in-office patient assessment). The LBPRS may also be useful because, despite the lack of information on SEM and MDC, the scale is quick and easy to use and allows an assessment of important aspects of the disease. Its usefulness in clinical research is also appreciated.

In summary, comparisons with other outcome measures suggest that each questionnaire has its advantages and may be appropriate for different contexts. The choice of questionnaire should take into account the specific needs of the study or clinical setting, such as the level of detail required and the target population. Overall, although each questionnaire has its own unique characteristics, together they contribute to a comprehensive understanding of disability in LBP. Further research is needed to explore the psychometric properties and applicability of these instruments in different populations and clinical scenarios.

## Figures and Tables

**Table 1:** Comparison of basic properties of selected scales.

	No of Items	Score Range	Answer Possibilities	General Result	Outcome Domains	Responsiveness (ROC) Curve	Reliability
ODI	10	0–100	Scaled text	lower score indicates better condition	pain interference and functioning (disease-specific)	0.76–0.89 [29,30,31]	Test–retest reliability values 0.73–0.99 [14,21,23,32] Internal consistency > 0.71 [14,21,23,32]
RMDQ	24	0–24	Yes/no	lower score indicates better condition	pain interference and disability level	0.75–0.84 [29,30,31]	Test–retest reliability values 0.83–0.91 [16,21,23]; Internal consistency > 0.84 [20,33]
QBPDS	20	0–100	6-point Likert scale	lower score indicates better condition	pain interference and functioning (disease-specific)	0.74–0.85 [31,34]	Test–retest reliability values 0.84–0.93 [21,35]; Internal consistency > 0.9 [21]
LBOS	13	0–75	Scaled text, 11-point VAS for pain	higher score indicates better condition	pain, functional, and ability items	values not reported	The test–retest reliability values 0.34–0.99 [36,37,38,39,40]; Internal consistency > 0.77 [36,37,38,39,40]
LBPRS	21	0–130	11-point VAS and 3-point Likert scale	lower score indicates better condition	pain, disability, and physical impairment	LBPRS_DISABILITY_ 0.6–0.95 LBPRS_PAIN_ 0.6–0.95 [29]	The test–retest reliability value 0.98 [28]; Internal consistency > 0.89 (sub-scores) Internal consistency > 0.96 (total scores) [29]

ODI—Oswestry Disability Index, RMDQ—Roland–Morris Disability Questionnaire, QBPDS—Quebec Back Pain Disability Scale, LBOS—Low Back Outcome Score, LBPRS—Low Back Pain Rating Scale.

## Data Availability

Not applicable.
